# Case report and discussion: Pre-implantation genetic diagnosis with surrogacy in vascular Ehlers–Danlos syndrome

**DOI:** 10.3389/fgene.2023.1147607

**Published:** 2023-03-16

**Authors:** Chloe Angwin, Neeti Ghali, Fleur Stephanie van Dijk

**Affiliations:** ^1^ London National Ehlers-Danlos Syndrome Service, North West Thames Regional Genetics Service, London North West Healthcare University NHS Trust, Harrow, United Kingdom; ^2^ Department of Metabolism, Digestion and Reproduction, Section of Genetics and Genomics, Imperial College London, London, United Kingdom

**Keywords:** pre-implantation genetic diagnosis, vascular, Ehlers–Danlos syndrome, *in vitro* fertilization, surrogacy

## Abstract

**Introduction:** Vascular Ehlers–Danlos syndrome (vEDS) is an autosomal dominant inherited connective tissue condition, characterized by generalized tissue fragility with an increased risk of arterial dissection and hollow organ rupture. In women with vEDS, pregnancy and childbirth carry significant risks of both morbidity and mortality. The Human Fertilisation and Embryology Authority has approved vEDS for pre-implantation genetic diagnosis (PGD), given the potential for life-limiting complications. PGD avoids implantation of embryos that are affected by specific disorders by carrying out genetic testing (either for a familial variant or whole gene) and selecting unaffected embryos prior to implantation.

**Case:** We present an essential clinical update to the only published clinical case of a woman with vEDS undergoing PGD with surrogacy, initially through stimulated in vitro fertilization (IVF) and in vitro maturation (IVM) and subsequently through natural IVF.

**Discussion:** In our experience, a subset of women with vEDS do wish to have biological, unaffected children through PGD despite being aware of the risks of pregnancy and delivery. Given the clinical heterogeneity in vEDS, these women could be considered on a case-by-case basis for PGD. Controlled studies with comprehensive patient monitoring evaluating the safety of PGD are essential to equitable healthcare provision.

## 1 Introduction

Pre-implantation genetic diagnosis (PGD) is available for many inherited conditions. Vascular EDS (vEDS), an inherited tissue condition characterized by generalized tissue fragility, is an accepted indication for PGD approved by the Human Fertilisation and Embryology Authority (HFEA) (HFEA, 2010). However, there is only one documented case of PGD in a woman diagnosed with vEDS (Bergeron et al., 2014); we present her medical history with an update on important clinical update on her reproductive history. In addition, the current European Society of Cardiology (ESC) guidance on pregnancy in cardiovascular disease patients and outcomes of pregnancy in women with vEDS will be discussed.

Ehlers–Danlos syndrome (EDS) is a heterogeneous group of connective tissue disorders characterized by joint hypermobility, skin and vessel fragility, and generalized tissue friability (Malfait, 2018). VEDS is a rare inherited connective tissue disorder that, in most cases, results from heterozygous pathogenic variants in the *COL3A1* gene encoding type III collagen (Malfait et al., 2017).

Major criteria for a diagnosis of vEDS consist of i) a family history of vEDS with a documented causative variant, ii) arterial rupture at a young age, iii) spontaneous sigmoid colon perforation in the absence of known pathology, iv) uterine rupture during the third trimester in the absence of previous C-section and/or severe peripartum tears, and v) carotid-cavernous sinus fistula formation in the absence of trauma (Malfait et al., 2017).

Depending on the specific underlying *COL3A1* gene variant, there will be decreased collagen type III production; this amount can vary from 50% to 87.5% (Malfait et al., 2020). Genotype–phenotype relationships have been observed with heterozygous protein-altering variants, accounting for earlier and more severe onset of symptoms than heterozygous null variants which cause a 50% reduction of collagen type III (Frank et al., 2015; Malfait et al., 2020). Intra and interfamilial variability have been noted in families with vascular EDS with regard to the age of onset and type of clinical events (Jørgensen et al., 2015).

For women with inherited disorders who wish to become pregnant, there are a variety of options including invasive prenatal diagnosis; however, only pre-implantation genetic diagnosis allows parents to have an unaffected, biological child without the risk of termination of pregnancy when the fetus is affected (NHS England, 2014). PGD is carried out through *in vitro* fertilization (IVF) of oocytes and spermatocytes from the parents, subsequent genetic testing of early embryos for the familial pathogenic variant(s), and selection of unaffected embryos *via* genetic testing (in this case of the *COL3A1* gene) prior to transfer to the uterus (NHS England, 2014). PGD does not screen embryos for other inherited conditions, and the fetus would be at a population risk of being affected by any other disorders (NHS England, 2014).

In women with vEDS, pregnancy does not only hold the 50% risk of having an affected child but also carries risk to maternal health including arterial events, uterine rupture, and death (5% risk of death) (Pepin et al., 2000; Byers et al., 2017; Malfait et al., 2017). In women affected by vEDS, these risks can be avoided through surrogacy. However, in surrogacy, the vEDS status of the fetus can still affect the pregnancy, for example, with increased rates of premature birth for fetuses affected by vEDS (Stephens et al., 2022). Surrogacy with PGD is an additional option where unaffected embryos can be transferred to an unaffected surrogate, reducing risk to the fetus and to the surrogate. However, altruistic surrogacy often requires expenses to be paid which may exclude this option for families and still carries the population-level risks of pregnancy and childbirth for the surrogate and fetus unaffected by vEDS (UK Government, 2021).

Current ESC guidance advises that pregnancy should be avoided in all women with vEDS, given the complication risks (European Society of Cardiology, 2018). However, there are no guidelines discussing assisted reproductive techniques in these women.

## 2 Case report

The original report (Bergeron et al., 2014) followed a 33-year-old woman, who had been diagnosed with vEDS due to a *de novo* heterozygous pathogenic *COL3A1* variant (c.2492G>A, p. Gly831Asp). She had a desire to have children while minimizing both the risk to herself from pregnancy-related complications and of her children inheriting the pathogenic variant and being affected with vEDS. We have detailed her medical history in the following paragraphs.

She was born with a unilateral congenital hip dislocation. At the age of 9, she suffered a left anterior cruciate ligament rupture while dancing. At 27, she had a hemorrhagic rupture of a liver cyst. The following year, she developed a right peroneal arterial aneurysm, which triggered genetic testing, and was molecularly confirmed to have vEDS. At age 29, she experienced a right coronary artery dissection complicated by a right iliac artery dissection post-angiogram.

At age 30, she started pre-conception counseling for PGD with surrogacy. Standard IVF protocols were commenced at age 32; however, during the first round of hormonal stimulation, egg retrieval was delayed over the weekend, and she had a splenic artery aneurysm rupture. This was her first exposure to IVF hormonal stimulation, and she was advised to avoid further doses after concerns regarding possible hormonal effects on vasculature. She developed a left-sided deep vein thrombosis (DVT) shortly after discharge, which was managed with standard anti-coagulation protocols, and developed a liver hematoma a month later. At the age of 33, she started natural (unstimulated) IVF and underwent four cycles with successful oocyte retrieval but unsuccessful implantation. After these cycles, she began the previously reported *in vitro* maturation (IVM) cycles and underwent six cycles with successful retrieval but unsuccessful implantation (Bergeron et al., 2014).

**FIGURE 1:**
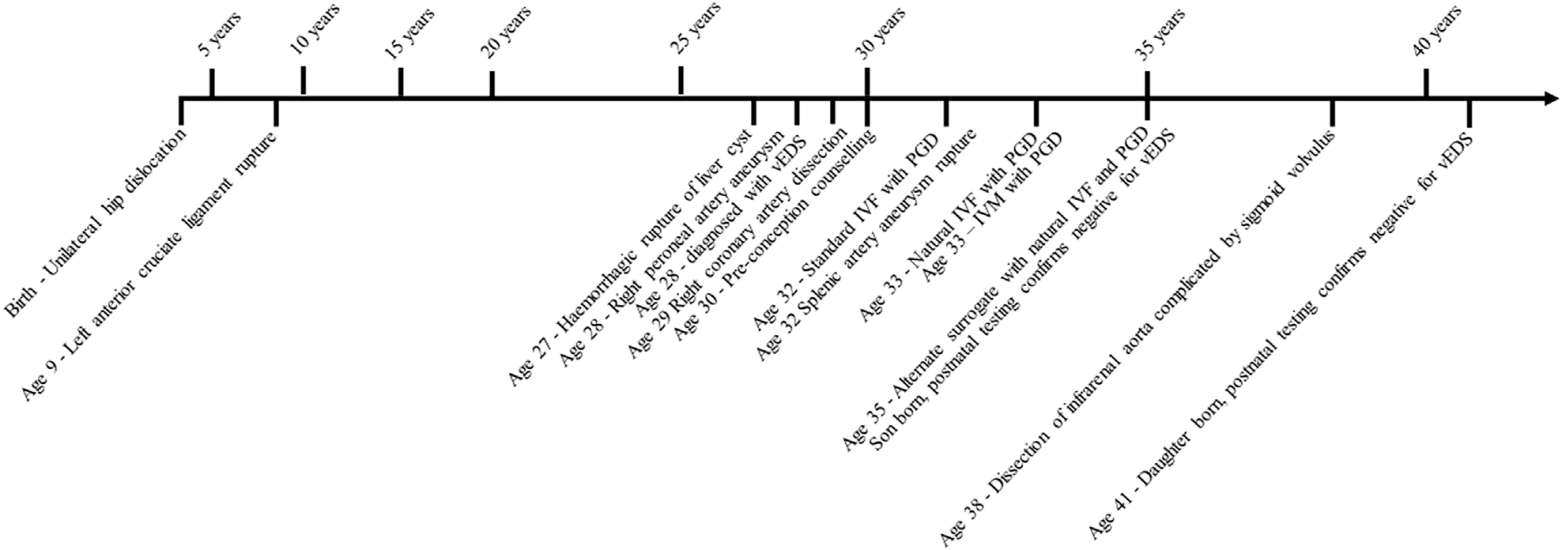
Timeline of clinical events recorded in the proband. IVF, *in vitro* fertilization; IVM, —*in vitro* maturation; vEDS, —vascular Ehlers–Danlos syndrome; PGD, —pre-implantation genetic diagnosis. Implantation and births *via* surrogate.

In addition to the publication of her medical history by Bergeron et al., a different surrogate was used and the patient continued natural IVF at age 35, undergoing six cycles to create four embryos. Implantation was successful, and her son was born and confirmed with prenatal testing and postnatal testing to be negative for the familial *COL3A1* variant. She continued natural IVF and underwent 20 cycles and 19 egg retrievals (after ovulating prior to one retrieval). All egg retrievals were transvaginal under sedation and carried out by a trained gynecologist. There was no reported organ damage or significant bleeds directly associated with the transvaginal egg retrievals. During this time, she had further vEDS-related complications; at age 38, she developed a spontaneous dissection of the infrarenal aorta below the inferior mesenteric artery complicated by sigmoid volvulus which was managed endoscopically. At age 41, another implantation was successful with the same surrogate; her daughter was born and confirmed to be negative for the familial *COL3A1* variant during prenatal and postnatal genetic testing (Figure 1).

## 3 Discussion

The reported patient, who had experienced a number of arterial events throughout her lifetime, underwent IVF with PGD and surrogacy and, as a result, had two children unaffected by vEDS. This involved a single cycle of hormonally stimulated IVF, followed by vascular complications, then four natural (without hormonal stimulation) IVF cycles and six IVM (*in vitro* maturation, collection, and maturation of oocytes *in vitro*) cycles without complication, and finally, 26 cycles of natural IVF with vascular complications midway through these cycles, unrelated to any procedures. As the only report in the literature of assisted conception in a woman with a molecularly confirmed diagnosis of vEDS, this report is relevant for a number of reasons. First, assisted conception and surrogacy could be considered a reproductive option for women with vEDS. Second, the sequence of serious arterial events prior to and following her single cycle of stimulated IVF questions the hypothesis that the first arterial event occurred purely due to ovarian hyperstimulation. Third, this patient underwent multiple (*n* = 33) transvaginal egg retrievals without complications, suggesting that this procedure may cause relatively low risks in women with vEDS. However, further investigations and formal studies are required to fully evaluate the risk of assisted conception in individuals with vEDS.

There is some evidence that hormonal treatments can exacerbate vascular fragility, alter the hemodynamic state, and precipitate cardiovascular events in susceptible patients (Iyasere and Potdar, 2022). Natural IVF involves close monitoring of natural ovulation cycles with no exogenous hormonal treatments but still requires manual egg retrieval.

General recommendations from the ESC regarding IVF in women with any cardiovascular disease include careful consideration of hormone dosages to manage or avoid prothrombotic conditions such as ovarian hyperstimulation syndrome and consideration of natural IVF (European Society of Cardiology, 2018). The ESC guidance makes no specific recommendations regarding egg retrieval risks or surrogacy in cardiovascular disease (European Society of Cardiology, 2018). General recommendations for cardiologists from the ESC advise that pregnancy is avoided in all women with a vEDS diagnosis (European Society of Cardiology, 2018). However, in reality, many female patients with vEDS continue to have pregnancies and are offered regular surveillance throughout. The lack of published data on PGD with or without surrogates in women with vEDS is important, given that there are women with vEDS who are aware of their diagnosis before they start a family and that vascular EDS is an accepted indication for PGD. Women with vEDS may potentially be discouraged from PGD as the risk from assisted reproductive techniques is unknown and pregnancy is deemed too risky. The most recent data on risks in pregnancy for women with vEDS are presented in the following paragraphs; however, it is important to note that not all individuals were diagnosed prior to pregnancy, and use of monitoring and current treatments (which are improving the life expectancy for this patient group) was not consistent (Frank et al., 2019).

Data were analyzed from 526 women with vEDS as an update to a previous cohort (Pepin et al., 2000), comparing 243 nulliparous women against a cohort of 283 women who had had at least one pregnancy (Murray et al., 2014). Women with vEDS had a pregnancy-related death rate of 4.9%. When stratified for the variant type, protein-altering variants resulted in a 5.3% death rate per delivery (30 deaths in 256 women across 565 delivered pregnancies), while there were no deaths in those women with heterozygous null variants. Importantly, Kaplan–Meier survival curve analysis showed no significant difference in survival between nulliparous and parous women with vEDS (Murray et al., 2014). In contrast, pregnancy-related mortality in the United States in 2017 was 0.02% across the entire population (Centres for Disease Control and Prevention, 2020). Within this study, a smaller cohort of 38 women with vEDS were interviewed about pregnancy-related complications; only 10.6% had a diagnosis of vEDS prior to conception, and three had null variants. Patients with null variants had a preterm delivery and third-degree and fourth-degree lacerations (Murray et al., 2014). The 35 women with protein-altering variants delivered 76 pregnancies, of which 49% (*n* = 35) of planned deliveries were uncomplicated. Complications included maternal death (*n* = 5), non-fatal vEDS-related complications (*n* = 5) (e.g., coronary artery dissection), and other complications (*n* = 46) including preterm delivery, third- or fourth-degree lacerations (only seen in vaginal deliveries), hemorrhage, and placenta previa or abruption (Murray et al., 2014).

The Registry of Pregnancy And Cardiac disease (ROPAC) has released data on pregnancy in women with thoracic aortic disease. This group contained four individuals with vEDS, one of whom had had a previous type B aortic dissection; all vEDS individuals underwent caesarean sections without complication (Campens et al., 2021).

Specific variants in *COL1A1* and *COL3A1* can result in overlapping phenotypes between classical EDS (thought to have a lower risk of vascular events) and vascular EDS. A recent study of 26 pregnancies in individuals with these variants reported no severe pregnancy-related complications (six perineal tears and one multiple miscarriage) (Colman et al., 2022). However, arterial events have been reported in this cohort during pregnancy, including a brachial artery dissection at 26 weeks in an individual with an atypical *COL3A1* variant (Ghali et al., 2019). Haploinsufficient variants in vEDS are thought to result in a milder phenotype, and a recent report of two haploinsufficient individuals did not identify any pregnancy-related complications (Kempers et al., 2022). Variation in phenotype severity may mean that pregnancy-related risk is also variable and could be assessed on a case-by-case basis (Frank et al., 2015; Jørgensen et al., 2015; Malfait et al., 2020).

Investigation of the frequency of complications related to fetal vEDS status found that premature birth was more commonly seen in affected rather than unaffected fetuses and that this was not impacted by the maternal vEDS status (Stephens et al., 2022). PGD with or without surrogacy, therefore, shows potential to reduce both maternal and fetal risks.

An alternative to PGD which ensures an unaffected child is invasive prenatal diagnosis (IPD), which would involve termination of any affected fetus (NHS England, 2014). ESC guidance discusses termination of pregnancy and recommends that for any women with high-risk cardiovascular disease, surgical termination in an experienced center is more appropriate, and medical terminations should be considered only up to 9 weeks using reduced dosages (European Society of Cardiology, 2018). However, prenatal testing can only be performed accurately after 11 weeks (Alfirevic et al., 2017). In women with vEDS, non-invasive prenatal diagnosis (NIPD) is currently not possible as the maternal variant will prevent differentiation from the fetus (Jenkins et al., 2018). Therefore, women with vEDS would have the option of a surgical termination only, which, given their generalized tissue fragility, would be considered a high-risk procedure. Additionally, the procedure of prenatal diagnosis is not without risk, particularly in the context of disorders of tissue fragility, and is also not acceptable to some individuals. Together, these concerns make prenatal diagnosis a potentially risky route and may discourage women with connective tissue disorders and vascular involvement from seeking prenatal genetic diagnosis as it would only be offered to those who would consider termination (a 50% risk for a woman with vEDS). There are no current published data on the safety of termination in women with vEDS.

In our experience, many women with vEDS remain keen to have their own biological children. Lack of communication regarding reproductive options and safety may put this group of patients at further risk as their ability to make an informed decision around reproduction is reduced and may, at worst, result in an unmonitored pregnancy. This is highlighted by the vEDS Research Collaborative members, who have recently published a research agenda which has pregnancy management as one of four important areas of focus (Sage et al., 2020).

## 4 Conclusion

We think that there is a place for considering each woman with vEDS for PGD with or without surrogacy on an individual basis, including medical history regarding events related to vEDS, family history, and specific underlying genetic cause. It is important to ensure that all women with vEDS who wish for biological children are counseled, informed of up-to-date risks, and have a clear understanding regarding the advantages and disadvantages of all available options including natural and hormonal IVF, PGD, and surrogacy.

We hope for a discussion regarding reproduction in women with vEDS despite the increased risk of death or complications in pregnancy in comparison to the general population. PGD offers the only route for families wishing to have unaffected biological children without the 50% risk of termination of pregnancy; if couples are well-informed of the hypothetical and potential risks, it may be that this could be offered on an individual case-by-case basis. Controlled studies with comprehensive patient monitoring evaluating the safety of PGD in these women are essential to equitable healthcare provision and patient counseling.

## Data Availability

The original contributions presented in the study are included in the article/Supplementary Material; further inquiries can be directed to the corresponding author.
